# A high-concentrate diet induced colonic epithelial barrier disruption is associated with the activating of cell apoptosis in lactating goats

**DOI:** 10.1186/s12917-014-0235-2

**Published:** 2014-09-26

**Authors:** Shiyu Tao, Yongqian Duanmu, Haibo Dong, Jing Tian, Yingdong Ni, Ruqian Zhao

**Affiliations:** Key Laboratory of Animal Physiology & Biochemistry, Ministry of Agriculture, Nanjing Agricultural University, Nanjing, Jiangsu China

**Keywords:** LPS, Apoptosis, Tight junctions, Colonic mucosa, HC diet, Goat

## Abstract

**Background:**

In ruminants, lower ruminal pH causes massive disruption of ruminal epithelial structure during periods of feeding high-concentrate diets. However, the influence of excessive organic fatty acids in the lumen of hindgut on the epithelial structure is unclear. In this study, twelve mid-lactating goats were randomly assigned to either a HC diet group (65% concentrate of dry matter; n = 6) or a LC diet group (35% concentrate of dry matter; n = 6) for 10 weeks. The colonic epithelial structure was detected by HE staining and transmission electron microscopy (TEM), and the apoptotic status of epithelial cells was estimated by TUNEL method and caspase activities.

**Results:**

HC goats showed higher level of free lipopolysaccharide (LPS) in rumen fluid (*p < 0.01*) but not in colonic digesta (*p > 0.05*), and higher total volatile fatty acid (VFA) concentrations in rumen fluid (*p < 0.05*) and in colonic digesta (*p < 0.01*), and higher content of starch in colonic digesta (*p < 0.05*) compared to LC goats. HC goats demonstrated profound alterations in the colonic epithelial structure and tight junctions (TJ), apparently due to damage of the epithelium with widened TJs space and nuclear breakdown and mitochondrial swelling. HC goats showed higher level of apoptosis in the colonic epithelium with higher proportion of TUNEL-positive apoptotic cells and increases of caspase-3 and −3/7 activities, as well as the lower ratio of bcl-2/bax mRNA expression in the colonic mucosa (*p < 0.05*). However, β-defense mRNA was significantly down-regulated in the colonic mucosa of HC goats compared to LC (*p < 0.05*). HC goats showed higher level of TJ proteins including claudin-1 and claudin-4 in the colonic mucosa than LC (*p < 0.05*). Neither free LPS content in the colonic digesta nor NF-κ B protein expression in tissues showed significant difference between HC and LC goats (*p > 0.05*).

**Conclusions:**

Our results reveal that long-term feeding HC diet to lactating goats causes severe damages to the colonic mucosa barrier associated with activating cells apoptosis.

**Electronic supplementary material:**

The online version of this article (doi:10.1186/s12917-014-0235-2) contains supplementary material, which is available to authorized users.

## Background

In current intensive production system, large amounts of cereal grains or easily degradable byproducts in diet are fed to lactating cows to meet energy requirement for supporting maintenance and high milk yields. Excessive amounts of rapidly fermentable nonstructural carbohydrates increase the accumulation of organic acids and shift of microbial population in gastrointestinal (GI) tract in ruminants [1,2]. It’s well documented that feeding high amounts of concentrate diet to ruminants results in subacute ruminal acidosis (SARA), a common metabolic disease especially occurred in high-producing animals. Lower pH due to the increase of short-chain fatty acids including lactic acid and volatile fatty acids (VFA) in rumen and higher endotoxin production derived from Gram-negative bacteria lead to the sever damages to rumen epithelium during SARA or acute ruminal acidosis. Previous studies mainly focused on the effects of feeding high-grain diet on the histological structure and functions of ruminal epithelium in dairy cows [3,4]. Compared to rumen epithelium with a stratum corneum layer and multicellular layers in the middle [5-7], the large intestine epithelium is much more “leaky” due to the monolayer structure [8]. In addition, the natural defense against ruminal acidosis such as saliva bicarbonate and Protoroa organisms with capacity of slowing pH down is lacking in the hindgut [9]. Therefore, differences in buffering capacity and histological structure between the rumen and hindgut make the hindgut less capable of maintaining digesta pH and microbial population during times of increased VFA production [10].

pH value is an extremely important factor to determine the status of epithelial barrier. Lan *et al.* reported that in HT-29 human colon adenocarcinoma cell line, short-chain fatty acids (SCFA) treatment in the pH range of 6.0 to 7.0 induced cell apoptosis rather than necrosis, while SCFA treatment at pH 5.5 caused cell necrosis [11]. Higher acidity induces apoptosis and inhibits cell proliferation in colorectal carcinoma cell lines [12]. In ruminants, high-grain diet led to an increase of acidity in intestinal tract, and the detrimental intestinal tract environment may cause apoptosis in the gut epithelium [10]. It’s well documented that several pathways are involved in cell apoptotic programs. One is mediated by the formation of the death-inducing signaling complex and activation of caspase-8 and caspase-10, two initiator caspases that in turn activate downstream effector of caspase-3 [13]. Another is mediated by proapoptotic signals at the mitochondria level including B-cell lymphoma 2 (Bcl-2) family and caspase-9 [14]. In epithelial tissues, tight junction damage or disruption is usually thought of as a downstream consequence of caspase cleavage during the apoptotic process [15].

As a physical barrier, intestinal epithelial mucosa separates the toxic compounds from the deeper intestinal layers [16,17]. Toll-like receptors (TLRs) in the gut epithelium play a key role in maintaining the homeostasis by recognizing ligands known as microbial-associated molecular patterns (MAMPs) derived from both pathogenic and non-pathogenic bacteria [18-20]. After combining with TLR-4 on the host cell surface, LPS activates myeloid differentiating factor 88 (MyD88), and then elicits a pro-inflammatory NF-κ B-dependent signaling cascade [21,22]. As a major transcription factor and a first responder to harmful cellular stimuli, NF-κB plays a central role in inflammation through its ability to induce transcription of pro-inflammatory genes [23]. Diverse stimuli (e.g., microbial products, microbes, pro-inflammatory cytokines, and oxidative stress) can activate NF-κB, and the downstream cytokines have been used to assess inflammation [24,25]. NF-κB activation has been detected in the mucosa of patients with inflammatory bowel disease (IBD) and in murine colitis model, and inhibition of NF-κB with a specific p65 antisense oligonucleotide is effective in preventing experimental models of IBD and efficiently down-regulates cytokine production [26]. It’s reported that the accumulation of free LPS in epithelial lumen will damage the integrity and permeability of epithelial barrier in rumen as well as in large intestine [8]. However, to our knowledge, after feeding high-grain diet for a long-term the changes of LPS signaling cascade in the hindgut mucosa of ruminants are still unknown.

Tight junctions (TJs) play an important role in maintaining the polarity of epithelial cells, regulating the permeability of the epithelial barrier and preventing the translocation of LPS and other toxic compounds from intestinal tract into circulating system [5,6]. To date, information regarding the effect of feeding ruminants with diet enriched high level of concentrate on epithelial structure in the hindgut is not available. Therefore, the objectives of this study were to investigate the changes of histological structure and ultrastructure of the colonic mucosa, and the status of epithelial cells apoptosis in mid-lactating goats fed a high concentrate diet for a long period.

## Methods

### Animals and experimental procedures

Twelve mid-lactating goats with approximately 49.7 ± 5.5 kg body weight were used in this study. Two weeks before the start of this experiment, goats were offered free access to a diet containing a forage-to-concentrate ratio (F: C) of 65:35 to ensure adaptation to the diet. After dietary adaptation, goats were randomly allocated to two groups. One group was fed a control diet comprising 65% forage and 35% mixed concentrate (low concentrate group, LC), while the other group received a high-grain diet containing 65% mixed concentrate and 35% forage (high concentrate group, HC). The details of the diet components and nutrient compositions were shown in Table 1. The animals were fed the respective diets for 10 weeks, and had free access to water during the experimental period.

**Table 1:** **Ingredients and composition of the experimental diets (%)**

**Items**	**The ratio of concentrate to forage**	
	**35:65**	**65:35**
Ingredients (% of DM)		
Leymus chinensis	52.0	28.0
Medicago sativa hay	13.0	7.0
Corn	25.6	25.0
Wheat bran	0	30.7
Soybean meal	7.4	2.2
Rape seed meal	0	4.0
Limestone meal	0.5	1.5
Calcium phosphate dibasic	0.8	0.7
Salt	0.4	0.4
Premix^1^	0.4	0.5
Total	100	100
Nutrient levels^2^ (%)		
Net energy(MJ/kg)	5.16	5.78
Digestible crude protein	7.31	8.05
Crude protein	12.17	13.42
Neutral detergent fiber	34.76	39.06
Acid detergent fiber	22.85	21.99
Calcium	0.72	1.04
Phosphorus	0.35	0.53

^1^Provided per kg of premix: Vitamin A 6 000U; Vitamin D2 500U; Vitamin E 80 mg; Cu 6.25 mg; Fe 62.5 mg; Zn 62.5 mg; Mn 50 mg; I 0.125 mg; Co 0.125 mg; Mo 0.125 mg.

^2^Nutrient levels were estimated from the current goat foods.

The protocol for the care, handling and use of animals followed the ARRIVE guidelines and was approved by Animal Ethics Committee at Nanjing Agricultural University, China. The sampling procedures complied with the “Guidelines on Ethical Treatment of Experimental Animals” (2006) No. 398 set by the Ministry of Science and Technology, China and “the Regulation regarding the Management and Treatment of Experimental Animals” (2008) No. 45 set by the Jiangsu Provincial People’s Government.

The ARRIVE guidelines was provided as Additional file 1.

### Samples collection

After 10 weeks feeding, goats were slaughtered after overnight fasting. Immediately after slaughter, the abdominal cavity was opened by midline incision, after that the rumen and intestinal tract were carefully removed. The rumen was opened from the dorsal side and rumen fluid was collected and strained through four layers of cheesecloth and kept on ice until processing. Digesta from the proximal colon was aseptically collected and kept on ice until being stored at −20°C. Within 20 min after slaughter, a segment of the colon wall from the same position of each animal was collected and the conlonic epithelium was separated from the muscular layers by blunt dissection and immediately washed three times in ice-cold phosphate buffered saline (PBS buffer). The tissue samples were frozen immediately in liquid nitrogen, and then used for extracting RNA and proteins.

### Rumen fluid sampling and assay

The rumen fluid was collected and divided into 2 portions. The first portion of each sample was transferred into a 50-mL sterile tube and kept on ice until transported to the laboratory for the initial processing before LPS determination as described by [27]. Briefly, rumen fluid samples were centrifuged at 10,000 × g for 45 min at 4°C and the supernatant was aspirated gently to prevent its mixing with the pellet and passed through a disposable 0.22-μm LPS-free filter. The filtrate was collected in a sterile glass tube (previously heated at 180°C for 4 h) and heated at 100°C for 30 min. Samples were cooled at room temperature (25°C) for 10 min and stored at −20°C for LPS analysis. The second portion of each rumen fluid sample was centrifuged at 3,000 × g for 15 min at 4°C immediately after collection and the supernatant was collected. To analyze VFA in ruminal fluid, a 5-mL aliquot was deproteinized with 1 mL of 25% metaphosphoric acid. These samples were stored at −20°C until analysis.

The concentration of LPS in rumen fluid was measured by a Chromogenic End-point Tachypleus Amebocyte Lysate Assay Kit (Chinese Horseshoe Crab Reagent Manufactory Co. Ltd, Xiamen, China). Pretreated rumen fluid samples were diluted until their LPS concentrations were in the range of 0.1 to 1 endotoxin units (EU)/mL relative to the reference endotoxin, and assayed as described by [27]. VFA were measured using capillary column gas chromatography (GC-14B, Shimadzu, Japan; Capillary Column: 30 m × 0.32 mm × 0.25 mm film thickness; Column temperature = 110°C, injector temperature = 180°C, detector temperature = 180°C).

### Colonic digesta sampling and assay

Colonic digesta samples were mixed thoroughly with an equal amount of physiological saline (0.90% wt/vol of NaCl). The mixtures were immediately centrifuged at 3,000 × g for 15 min and the supernatants were stored at −20°C until analyzed for LPS and VFA detection. 10 g of sample was transferred into a pyrogen-free tube with 10 mL of physiological saline and mixed vigorously. Samples were then processed and analyzed for LPS and VFA using the same procedure described earlier for rumen fluid samples. The LPS concentration in colonic digesta samples was expressed as endotoxin units (EU) per gram of wet sample.

Colonic digesta samples were dried at 60°C for 48 h. Dried samples were subsequently ground using a Wiley mill through a 1-mm screen (Thomas-Wiley, Philadel-phia, PA) and stored at −20°C until analyzed for starch using a Total Starch assay kit (Comin Biotechnology Co. Ltd, suzhou, China).

### Caspase-3 and −3/7 activity assay

Caspase-3 enzyme activity of the colonic mucosa tissue was measured by caspase activity Assay Kit (Jiancheng Bioengineering Institute, nanjing, China). And caspase-3/7 enzyme activity Assay Kit was purchased from Sigma (St. Louis, MO, USA). The procedures were performed according to the manufacture’s instruction.

### Histopathology, transmission electron microscopy and TUNEL

Specimens of the intestinal wall of the colonic mucosa were prepared for histological examination by fixing in 4% formaldehyde-buffered solution, embedding in paraffin, and sectioning. Specimens were examined for injury after hematoxylin and eosin (H&E) staining as described by [28].

Colonic mucosa tissue samples were separated and fixed immediately with 2% glutaraldehyde, post-fixed with 1% osmium tetroxide, and embedded in resin. Ultrathin sections were cut and stained with uranyl acetate and lead citrate. Epithelial tissues ultrastructure was determined with a transmission electron microscope (Hitachi H-7650, Hitachi Technologies, Tokyo, Japan).

Apoptotic epithelial cells in colonic tissue were analyzed using the terminal deoxynucleotidyl transferase (TdT)-mediated dUTP-biotin nick end labeling (TUNEL) assay according to the manufacturer’s instruction. Apoptosis detection kit was supplied by Boster Bio-engineering limited company (Wuhan, China). TUNEL-positive nuclei were clearly identified as brown-stained nuclei, which indicated the presence of DNA fragmentation due to apoptosis. TUNEL-positive cells were determined by observing 1000 cells in randomly selected fields.

### RNA isolation, cDNA synthesis and real-time PCR

Colonic mucosa tissue was quickly collected and immediately frozen in liquid nitrogen, and stored at −80°C until RNA isolation. Total RNA was extracted from colon samples with Trizol Reagent (15596026, Invitrogen). Concentration and quality of the RNA were measured by NanoDrop ND-1000 Spectrophotometer (Thermo, USA). Then two micrograms of total RNA were treated with RNase-Free DNase (M6101, Promega, USA) and reverse-transcribed according to manufacturer’s instructions. Two microliter of diluted cDNA (1:40, vol/vol) was used for real-time PCR which was performed in Mx3000P (Stratagene, USA). GAPDH, which is not affected by the experimental factors, was chosen as the reference gene. All the primers chosen to study the expression of genes related to TJs and apoptosis, as listed in Table 2, were synthesized by Generay Company (Shanghai, China). The method of 2^-△△Ct^ was used to analyze the real-time PCR results and gene mRNA levels were expressed as the fold change relative to the mean value of control group.

**Table 2:** **PCR primer sequences of the target genes**

**Target genes**	**Reference/Genbank accession**	**PCR products (bp)**	**Primer sequences**
GAPDH	HM043737.1	180	F: 5’-GGGTCATCATCTCTGCACCT -3’
			R: 5’-GGTCATAAGTCCCTCCACGA -3’
Occludin	BC133617.1	200	F: 5’-GTTCGACCAATGCTCTCTCAG -3’
			R: 5’-CAGCTCCCATTAAGGTTCCA -3’
Claudin-1	HM117762.1	216	F: 5’-CACCCTTGGCATGAAGTGTA-3’
			R: 5’-AGCCAATGAAGAGAGCCTGA -3’
Claudin-4	HM117763.1	238	F: 5’-AAGGTGTACGACTCGCTGCT-3’
			R: 5’-GACGTTGTTAGCCGTCCAG-3’
β-defensin	HM593790.1	165	F: 5’- CTGCTGGGTCAGGATTTAC -3’
			R: 5’- GCGTCTTCGCCTTCTGTT -3’
Bcl-2	AY423861.1	208	F: 5’- TCGCCCAAGTCAAACATTA-3’
			R: 5’- CACAGGTGAAACTGCCAAGAT-3’
Bax	AF163774.1	178	F: 5’-TGCTCACTGCCTCACTCAC-3’
			R: 5’-CCAAGACCACTCCTCCCTA-3’

### Western blotting analysis

100 mg frozen colonic mucosa tissue was minced and homogenized in 1 mL of ice-cold homogenization buffer RIPA containing the protease inhibitor cocktail Complete EDTA-free (Roche, Penz-berg, Germany). The homogenates were centrifuged at 12,000 rpm for 20 min at 4°C and then collected the supernatant fraction. Protein concentration was determined using a BCA Protein Assay kit (Pierce, Rockford, IL, USA). Eighty micrograms of protein extract from each sample was then loaded onto 7.5% and 15% SDS-PAGE gels and the separated proteins were transferred onto the nitrocellulose membranes (Bio Trace, Pall Co, USA). After transfer, membranes were blocked for 2 h at room temperature in blocking buffer and then membranes were incubated with the following primary antibodies: rb-anti-NF-κB p65 (1:500; sc-372, Santa cruz), m-anti-occludin (1:500; 33–1500, Invitrogen), rb-anti-claudin-1 (1:200; sc-28668, Santa cruz), m-anti-claudin-4 (1:500; 32–9400, Invitrogen), rb-anti-actived-caspase-3 (1:500; BS7004, Bioworld) and GAPDH (1:10000; AP0066, Bioworld) in dilution buffer overnight at 4°C. After several times washes in Tris-Buffered-Saline with Tween (TBST), membranes were incubated with goat anti-rabbit or goat anti-mouse horseradish peroxidase (HRP)-conjugated secondary antibodies (1:10000; Bioworld, USA) in dilution buffer for 2 h at room temperature. Finally, the blot was washed and detected by enhanced chemiluminescence’s (ECL) using the LumiGlo substrate (Super Signal West Pico Trial Kit, Pierce, USA), and the signals were recorded by an imaging System (Bio-Rad, USA), and analyzed with Quantity One software (Bio-Rad, USA).

### Statistical analysis

Data are presented as means ± SE. Statistical significance was assessed by the independent sample t-test using SPSS (SPSS version 11.0 for Windows; SPSS Inc., Chicago, IL, USA) software packages. Data was considered statistically significant when *P* < 0.05. Numbers of replicates used for statistics are noted in the Tables and Figures.

## Results

### Volatile fatty acid (VFA) and LPS concentrations in rumen fluid and colonic digesta

Concentrations of propionate (*p < 0.01*), butyrate (*p < 0.01*), isobutyrate (*p < 0.05*), valerate (*p < 0.01*), isovalerate (*p < 0.001*) and total VFA (*p < 0.05*) in rumen fluid were significantly increased in HC goats compared to LC. The level of acetate (*p < 0.01*), propionate (*p < 0.05*), butyrate (*p < 0.01*) and total VFA (*p < 0.01*) concentrations in colonic digesta of HC goats was significantly higher than that of LC goats. As shown in Table 3, HC goats showed markedly higher level of free LPS concentration in rumen fluid than LC goats (*p < 0.01*), while there was no significant difference of free LPS concentration in colonic digesta between HC and LC goats (*p > 0.05*). Additionally, starch content in colonic digesta of HC goats was markedly higher than that in LC goats (*p < 0.05*) (Table 3).

**Table 3:** **The effect of feeding LC or HC diet on rumen fermentation and colonic digesta parameters in goats at the time of slaughter**

**Item**	**LC**	**HC**	**P-value**
**Rumen fluid**			
Free LPS, EU/mL	25201 ± 3398	48395 ± 4723	0.004
Total VFA, mM	90.20 ± 3.55	116.37 ± 8.14	0.023
Acetate, mM	58.28 ± 2.45	65.48 ± 5.45	0.291
Propionate, mM	16.14 ± 0.55	22.03 ± 1.24	0.003
Butyrate, mM	10.65 ± 0.77	21.36 ± 1.79	0.001
Isobutyrate, mM	1.73 ± 0.06	2.12 ± 0.14	0.044
Valerate, Mm	1.26 ± 0.08	1.92 ± 0.12	0.002
Isovalerate, mM	2.14 ± 0.06	2.95 ± 0.12	0.000
Acetate: Propionate	3.61 ± 0.12	2.96 ± 0.14	0.007
**Colon digesta**			
Free LPS,EU/mL	22527 ± 5325	33613 ± 5390	0.182
Starch, mg/g mass	3.42 ± 0.51	4.56 ± 0.93	0.042
Total VFA, mM	44.68 ± 3.35	59.01 ± 2.51	0.007
Acetate, mM	26.54 ± 1.97	36.58 ± 2.08	0.007
Propionate, mM	10.79 ± 0.88	13.23 ± 0.55	0.037
Butyrate, mM	4.44 ± 0.54	6.26 ± 0.25	0.010
Isobutyrate, mM	2.37 ± 1.46	0.85 ± 0.04	0.280
Valerate, Mm	1.07 ± 0.04	1.21 ± 0.06	0.130
Isovalerate, mM	0.95 ± 0.03	0.88 ± 0.03	0.172
Acetate: Propionate	2.47 ± 0.04	2.78 ± 0.16	0.126

Values are mean ± SEM, n = 6.

### Caspase activities in the colonic mucosa

**Figure 1:**
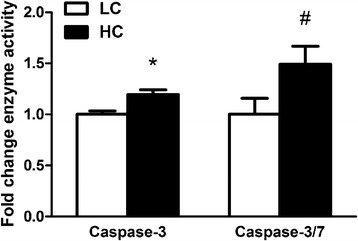
**Caspase-3 and caspase-3/7 enzyme activity in the colonic mucosa.** The results were expressed as mean ± SEM. The data were analyzed by Independent-Samples T test using the Compare Means of SPASS 11.0 for Windows (StaSoft Inc, Tulsa, OK, USA). *p < 0.05 vs. LC.

As shown in Figure 1, HC goats showed a significant increase of caspase-3 activity (*p < 0.05*) and a tendency increase of caspase-3/7 activity (*p = 0.07*) in colonic mucosa compared to LC.

### Morphology, ultrastructure and TUNEL of the colon epithelium

**Figure 2:**
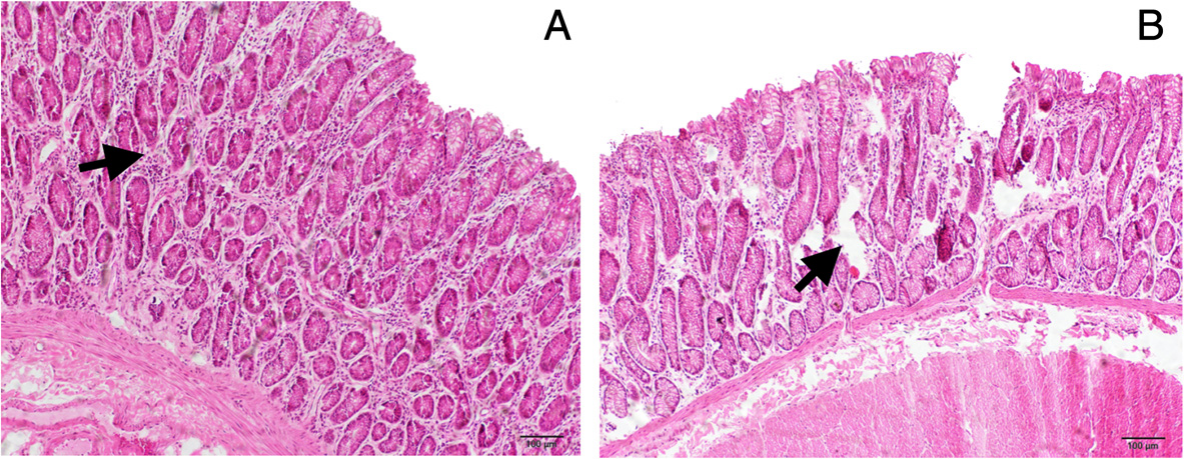
**Comparisons of morphological of the colonic mucosa between HC and LC goats.** Colonic mucosa epithelium (n = 6) from each group were processed for morphological evaluation: colon section of the (**A**, scale bar = 100 μm) LC group; (**B**, scale bar = 100 μm) HC group. Arrow indicates the damage of the colonic mucosa epithelium.

**Figure 3:**
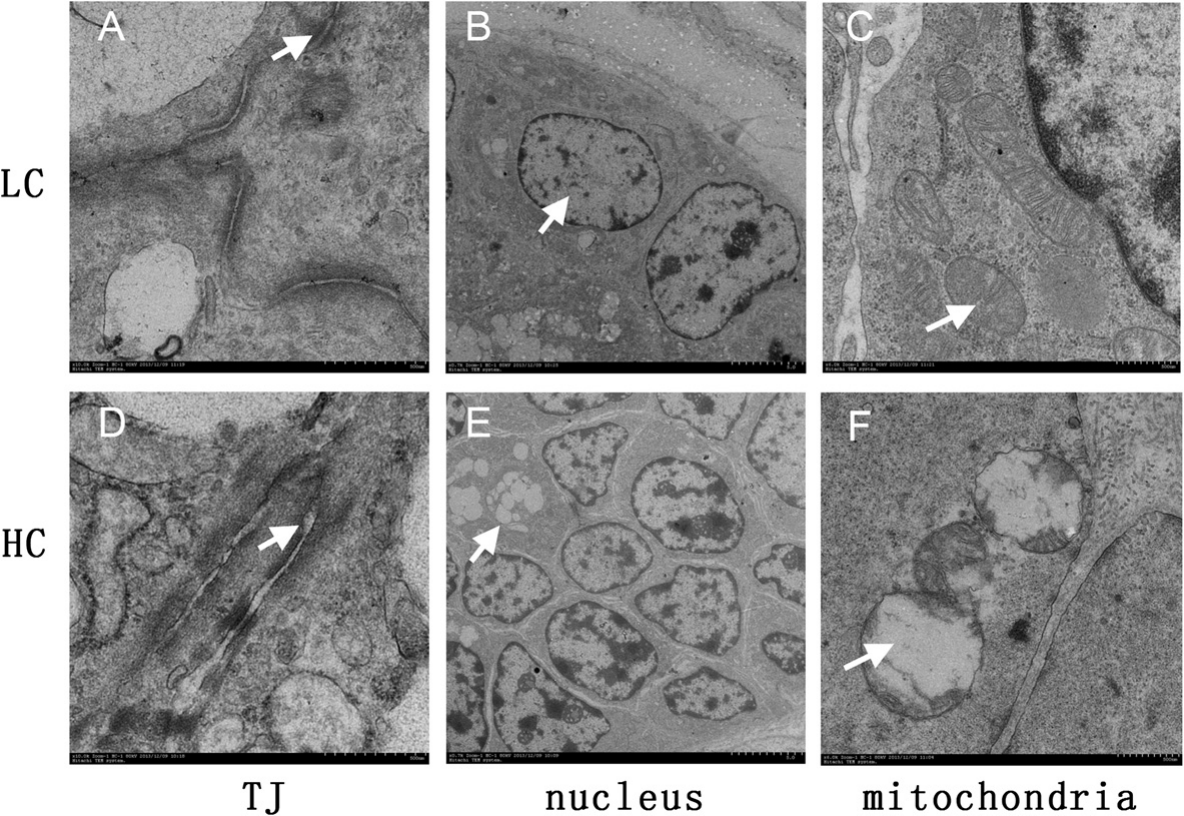
**Comparisons of ultrastructure of the colonic mucosa between HC and LC goats.** Colonic mucosa epithelium (n = 6) from each group were processed for ultrastructure evaluation: colon section of the **(A)** TJs of LC group; **(B)** nuclear of LC group; **(C)** mitochondria of LC group; **(D)** TJs of HC group; **(E)** nuclear of HC group; **(F)** mitochondria of HC group (transmission electron microscopy, × 10000). Arrow indicates the location of the TJs, nuclear or mitochondria (Scale bar = 500 nm).

**Figure 4:**
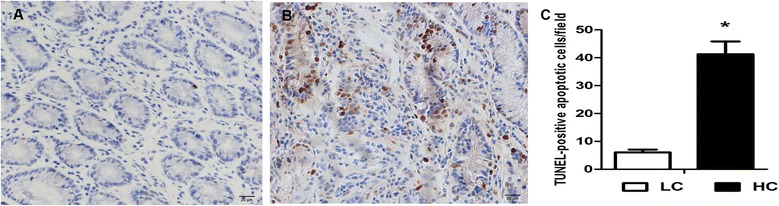
**Comparisons of TUNEL of the colonic mucosa between HC and LC goats.** Colonic mucosa epithelium (n = 6) from each group were processed for TUNEL-positive apoptotic cells evaluation: colon section of the (**A**, scale bar = 20 μm) LC group; (**B**, scale bar = 20 μm) HC group. **C**: Analysis of the positive apoptotic cells. The results were expressed as mean ± SEM. The data were analyzed by Independent-Samples T test using the Compare Means of SPASS 11.0 for Windows (StaSoft Inc, Tulsa, OK, USA). *p < 0.05 vs. LC.

HE staining showed that indentations, severe cellular damage and crypts necrosis were observed in the colonic epithelium of HC but not LC goats (Figure 2A and B). The TJ and epithelium apoptosis in the colonic epithelium was detected by Transmission Electron Microscopy (TEM) method. TJs in the colonic epithelium of HC goats were damaged with wider intercellular space (Figure 3A and D), while LC goats displayed integrity and normal TJs structure. Moreover, LC goats showed normal cell nucleus and mitochondria structure (Figure 3B and C), whereas HC goats displayed apparent nuclear breakdown and mitochondrial swelling (Figure 3E and F). As shown in Figure 4, the proportion of TUNEL-positive apoptotic cells in the colonic epithelium of HC goats was markedly increased compared to LC goats (*p < 0.05*).

### Gene expression in the colonic mucosa

**Figure 5:**
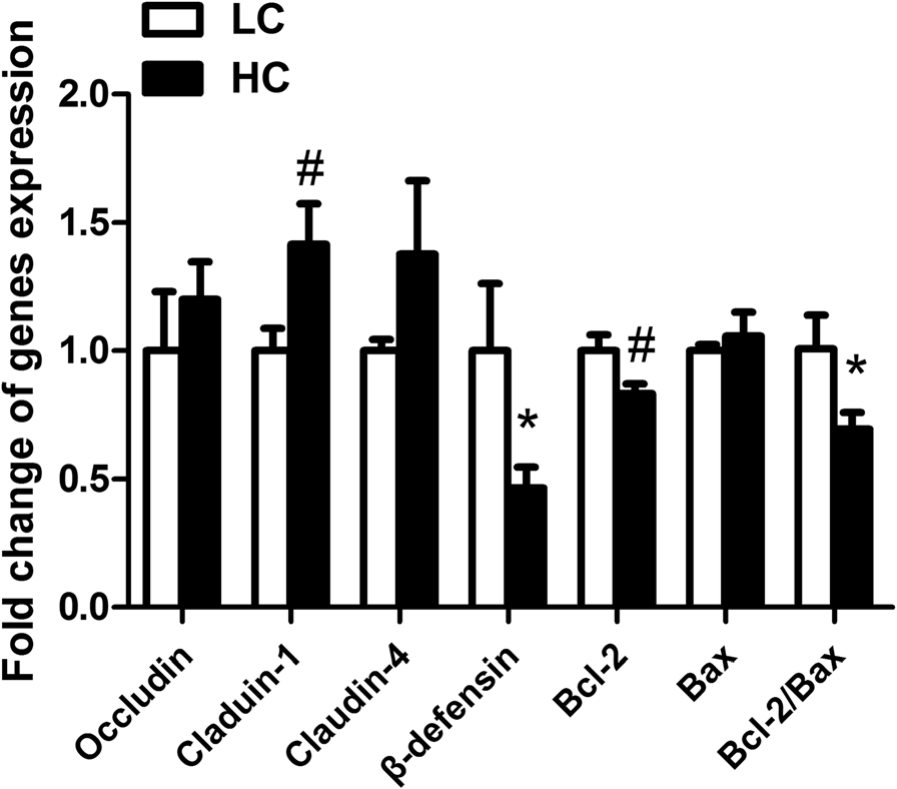
**Gene expression in the colonic mucosa.** GAPDH was used as the reference gene for gene expression. The data were analyzed by Independent-Samples T test using the Compare Means of SPASS 11.0 for Windows (StaSoft Inc, Tulsa, OK, USA). Values are mean ± SEM. ^*#*^ *p < 0.1*, *p < 0.05 vs. LC.

In the colonic mucosa, β-defensin and the ratio of bcl-2/bax mRNA expression was significant decreased in HC goats compared to control (*p < 0.05*). There was a tendency increase in mRNA expression of claudin-1 in HC goats (*p = 0.084).* However, the mRNA expression of occludin and claudin-4 in the colonic mucosa did not show significant difference between HC and LC goats (*p > 0.05,* Figure 5).

### Protein expression in the colonic mucosa

**Figure 6:**
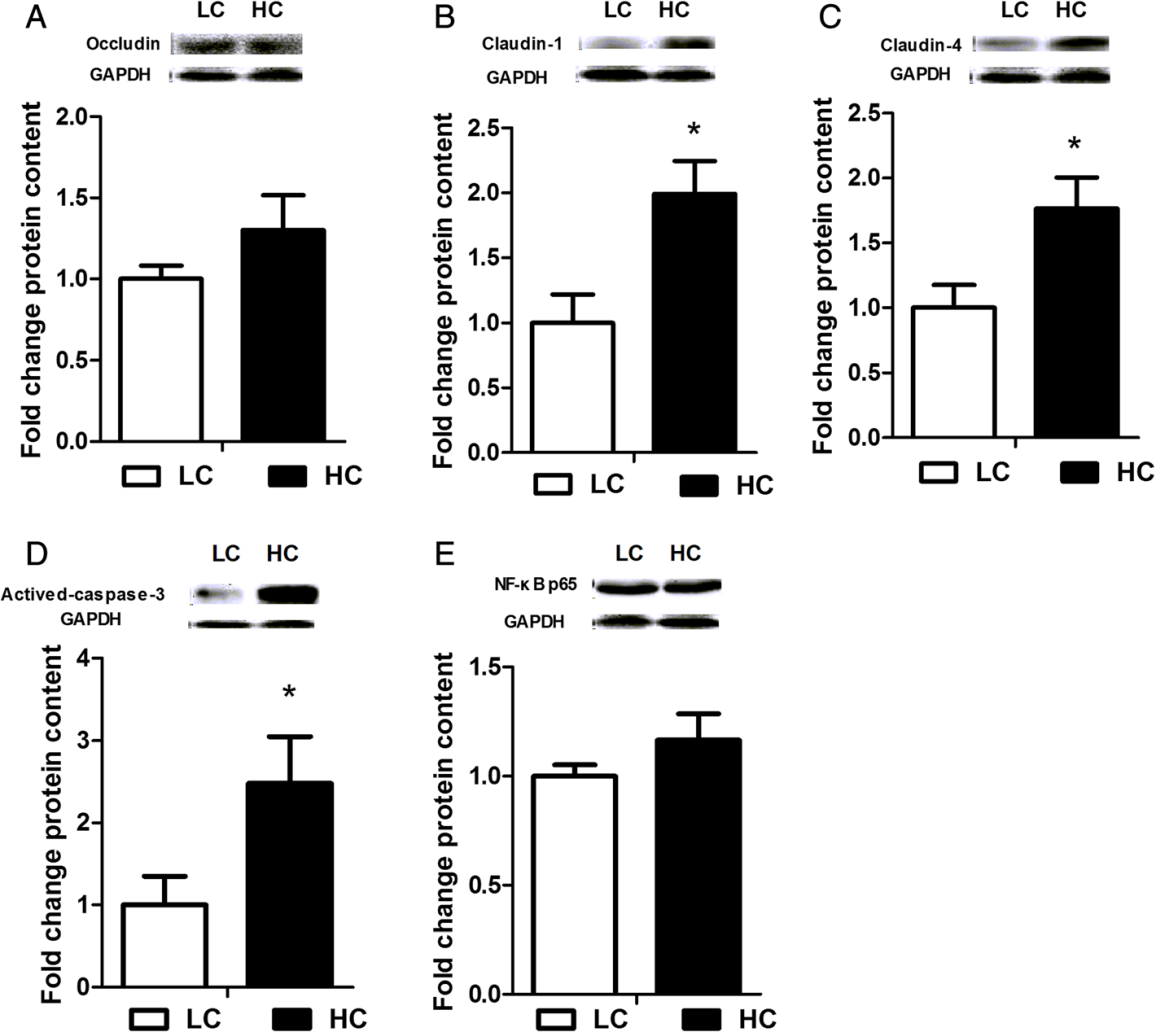
**Protein expression in the colonic mucosa.** Results of protein levels expressed as arbitrary units relative to GAPDH protein, fold change of Occludin **(A)**, Claudin-1 **(B)**, Claudin-4 **(C)**, Actived-caspase-3 **(D)** and NF-κB p65 **(E)** protein content in the colonic mucosa. Values are mean ± SEM. The data were analyzed by Independent-Samples T test using the Compare Means of SPASS 11.0 for Windows (StaSoft Inc, Tulsa, OK, USA). ^*#*^ *p < 0.1*, *p < 0.05 vs. LC.

The level of claudin-1 (*p < 0.05*), claudin-4 (*p < 0.05*) and actived-caspase-3 (*p < 0.05*) proteins expression in the colonic mucosa was significantly up-regulated in HC goats compared to LC. However, occludin and NF-κB proteins expression in the colonic mucosa was not altered by high concentrate diet treatment (*p > 0.05*) Figure 6.

## Discussion

Feeding high-grain diets to lactating ruminants causes a high risk to damage the histological integrity and functions of ruminal epithelium in dairy cows [3,4]. However, information regarding the influence of high-concentrate diet on the hindgut epithelial structure, and the molecular events especially the alterations in TJ proteins and epithelial cells apoptosis status is currently unavailable. The present study reports for the first time that feeding high proportion of concentrate diet to lactating goats for 10 wks increase VFA concentrations in both ruminal fluid and colonic digesta. The severe damages of the colonic mucosa barrier indicated by widen TJs space and nuclear breakdown and mitochondrial swelling, which was associated with the activating of epithelial cells apoptosis showing by the significant increase of TUNEL-positive apoptotic cells and caspases activities. These results may provide new insights into the understanding the relationship between abnormal fermentation in the hindgut lumen and the damages to the intestinal mucosa barrier.

After 10 wks feeding, HC-fed goats showed a higher level of total VFA content in ruminal fluid compared to LC goats. As previous report [10], our results showed that a significant increase of both total VFA and starch content in the colonic digesta observed in HC goats. It’s reported that a single mild episode of subacute ruminal acidosis (SARA) does not affect ruminal barrier function in the short term [29], and the author inferred that increased acid insult severity might induce sustained epithelial barrier dysfunction. In production practice, impairment of intestinal epithelial barrier function is frequently found in high-producing ruminants which are usually fed a highly concentrated diet for relatively long time. Paralleled the accumulation of VFA and starch in colonic digesta, a profound structural disruptions in the colonic mucosa was observed in HC goats with widen TJs, cell nuclear breakdown and mitochondrial swelling. In contrast, LC goats displayed the integrity of mucosa barrier with normal histological structure and cellular ultrastructure in the colonic epithelium. Our results demonstrate that, as previous reports in ruminal epithelial barrier [21,28], feeding high-concentrate diet to lactating goats for long period resulted in the disruption of epithelial structure in the colonic mucosa.

Luminal acidity is one of the most important factors to determine the status of epithelial barrier. It’s reported that acetic acid (0.1 M) showed a time- and pH-dependent ability to damage colonic epithelium in pig [30]. In human colon adenocarcinoma cell line, acetate treatment in the pH range of 6.0 to 7.0 induced cell apoptosis rather than necrosis, while acetate treatment at pH 5.5 caused cell necrosis [11]. In addition, higher acidity induces apoptosis and inhibits cell proliferation in colorectal carcinoma cell lines [12]. In this study, the level of acetate and other VFA components in the colonic digesta was markedly increased in HC goats. TUNEL results showed that in parallel to the obvious damages to the colonic mucosa, higher amount of positive apoptotic cells were detected in the colonic mucosa of HC goats than that of LC counterparts. Moreover, HC goats demonstrated a marked appearance of dark brown apoptotic cells and intercellular apoptotic fragments compared to LC goats. Previous studies suggested that the short-chain fatty acids increased localization epithelial apoptosis and necrosis and these changes are dependent on caspase activation [11]. In epithelial tissues, tight junction damage is usually thought of as a downstream consequence of caspase cleavage during the apoptotic process [15]. In the present study, the activities of caspase-3 and caspase-3/7 were markedly enhanced in the colonic epithelium of HC goats compared to control. In addition, the level of activated-caspase-3 protein was also enhanced in the colonic epithelium of HC goats. As the previous report [31], our results showed that the ratio of bcl-2/bax mRNA expression was significant decreased in HC goats compared to control, which indicates the down-regulation of the cellular anti-apoptotic ability in the colonic mucosa. Based on these results, we conclude that an increase of cellular apoptosis and a decrease in anti-apoptotic ability may contribute to the damages of the colonic mucosa caused by HC diet.

TJ proteins defects or enrichment are causatively associated with a variety of human diseases, demonstrating that TJ proteins play important roles in human physiology [16,32-34]. In this study, accompanied the disruption and expansion of intercellular TJs morphology, HC goats demonstrated a marked up-regulation of TJ protein expression including claudin-1 and −4 in the colonic mucosa. There are several possible explanations for these changes in TJ proteins. As previous reports [35,36], the claudins comprise a multigene family, and the different claudins have diverse functions depending on cell type and the host organism. It’s reported that the claudin-1 does not localize to the TJ, which indicates no contribution of claudin-1 to the barrier function [37]. Moreover, recent studies have revealed that claudins may be involved in regulating cell proliferation and signaling [38-41]. Claudin-4 has been suggested as one of these unique types of claudins, which strongly participated in membrane permeation via paracellular pathway in both normal and disease condition [42,43]. Claudin-4 expression was significantly increased in the intestinal mucosa lesion [39]. Another kind of TJ protein, Occludin has a transmembrane region and may play both a functional and structural role defining the paracellular barrier [44]. High-grain diet caused a significant change of occludin mRNA and protein expression in ruminal epithelium [25]. However, our data showed that there was no significant difference in the expression of occludin mRNA and protein in the colonic mucosa between HC and LC goats. Nevertheless, the true causal relationship between TJs disruption and the increase of claudins protein in the colonic mucosa of HC goats needs to be further elucidated.

As one of the most potent inflammatory mediators and a major structural component of Gram-negative bacteria, LPS has been hypothesized to form an important risk factor of intestinal bowel disease (IBD) [45]. It's widely accepted that Toll-like receptors (TLRs) in the intestine epithelium play a key role in maintaining the homeostasis by recognizing ligands known as microbial-associated molecular patterns derived from both pathogenic and non-pathogenic bacteria [18,19]. MyD88-dependent pathway is the downstream signals of TLR-4, and initiation of MyD88-dependent pathway could lead to activation of NF-κB and transcription of several pro-inflammatory genes [18]. NF-κB activation has been detected in the mucosa of patients with IBD and in murine colitis model, and inhibition of NF-κB with a specific p65 antisense oligonucleotide is effective in preventing experimental models of IBD and efficiently down-regulates cytokine production [26]. In ruminants, the increase in rumen LPS concentration due to increased starch feeding is well documented [10,46]. The increase in LPS concentration in the cecum in grain-based SARA challenges are due to increased growth of LPS-producing bacteria in the hindgut but not in the rumen [43]. Van Kessel *et al.* observed an increase of the gram-negative bacteria in cecal digesta after postruminal infusion of starch [47]. In this study, a significant increase of LPS in ruminal fluid was observed in HC goats, and starch content in colonic digesta was also markedly increased compared to LC goats. In a good agreement with previous studies, a significant increase of LPS in cecal digesta was observed in the present study (data not shown). However, LPS concentration in colonic digesta was not altered after feeding HC diet for 10 wks. The differences in adaptation time to diet and different hindgut location between these studies may explain the discrepancy results of LPS production in hindgut digesta. In order to further investigate the influence of LPS signal pathway on the epithelial cellular function, NF-κ B protein expression in the colonic mucosa was detected by western blot. No significant difference of NF-κ B protein was observed in the colonic mucosa between HC and LC goats. Taken together, these results suggested that LPS cascade signal may not contribute to the damages of the colonic mucosa induced by feeding HC diet for a long-term in lactating goats.

## Conclusion

In summary, we report herein for the first time that long-term feeding HC diet to lactating goats caused the accumulation of VFA in ruminal fluid and colonic digesta and damages to the colonic mucosa barrier induced by activating cells apoptosis. For HC goats, the increase of acidity rather than LPS in colonic digesta is mostly responsible for the disruption of the colonic epithelial barrier.

## Additional file

Additional file 1:
**Animal research: reporting in vivo experiments.**
