# Supplementary material for: A high-concentrate diet induced colonic epithelial barrier disruption is associated with the activating of cell apoptosis in lactating goats
Source: BMC Vet Res. 2014 Sep 26;10:235. doi: 10.1186/s12917-014-0235-2 (PMC4180839; doi:10.1186/s12917-014-0235-2)
Supplement: Additional file 1: — Animal research: reporting in vivo experiments. [file 12917_2014_235_MOESM1_ESM.docx]

**The ARRIVE checklist:**

**Animal Research: Reporting In Vivo Experiments**

Shiyu Tao^1^, Yongqian Duanmu^1^, Haibo Dong^1^, Jing Tian^1^, Yingdong Ni^1*^, Ruqian Zhao^1^

|  | Item |  |
| --- | --- | --- |
| Title | 1 | A high-concentrate diet induced colonic epithelial barrier disruption is associated with the activating of cell apoptosis in lactating goats. |
| Abstract | 2 | BACKGROUND AND PURPOSE: Previous studies mainly focused on the effects of feeding high-grain diet on the histological structure and functions of ruminal epithelium in dairy cows. Compared to rumen epithelium with a stratum corneum layer and multicellular layers in the middle, the large intestine epithelium is much more “leaky” due to the monolayer structure. Our aim was to evaluate the effects of feeding high concentrate diet for a long period on the histological structure and ultrastructure of the colonic mucosa, and the status of epithelial cells apoptosis in mid-lactating goats.  EXPERIMENTAL APPROACH: Twelve mid-lactating goats received a high-grain diet containing 65% mixed concentrate and 35% forage (HC). Control group received a low concentrate diet comprising 65% forage and 35% mixed concentrate (LC). After 10 weeks, we determined the colonic epithelial structure by HE staining and transmission electron microscopy (TEM), and the apoptotic status of epithelial cells by TUNEL method and Caspase activities. The level of mRNA expression of β-defensin, bcl-2, bax, claudins and occludin in the colonic mucosa was determined by real-time PCR. The content of claudins, occluding, NF-κB and actived-caspase-3 in the colonic mucosa homogenate was performed by western blot.  KEY RESULTS: We have demonstrated that long-term feeding HC diet to lactating goats causes severe damages to the colonic mucosa barrier; and these effects could be attributed to the activating cells apoptosis.  CONCLUSIONS AND IMPLICATIONS: These results highlighted the negative effects of long-term feeding HC diet on the hindgut mucosa barrier and suggest the importance of feeding high proportion of high quality forage diets to lactating ruminants. |
| INTRODUCTION |  |  |
| Background | 3 | 1. Excessive amounts of rapidly fermentable nonstructural carbohydrates increase the accumulation of organic acids and shift of microbial population in gastrointestinal (GI) tract in ruminants [[1](#_ENREF_1), [2](#_ENREF_2)]. Lower pH due to the increase of short-chain fatty acids including lactic acid and volatile fatty acids (VFA) in rumen and higher endotoxin production derived from Gram-negative bacteria lead to the sever damages to rumen epithelium during SARA or acute ruminal acidosis. Previous studies mainly focused on the effects of feeding high-grain diet on the histological structure and functions of ruminal epithelium in dairy cows [[3](#_ENREF_3), [4](#_ENREF_4)]. Compared to rumen epithelium with a stratum corneum layer and multicellular layers in the middle [[5-7](#_ENREF_5)], the large intestine epithelium is much more “leaky” due to the monolayer structure [[8](#_ENREF_8)]. In addition, the natural defense against ruminal acidosis such as saliva bicarbonate and Protoroa organisms with capacity of slowing pH down is lacking in the hindgut [[9](#_ENREF_9)]. Therefore, differences in buffering capacity and histological structure between the rumen and hindgut make the hindgut less capable of maintaining digesta pH and microbial population during times of increased VFA production [[10](#_ENREF_10)]. pH value is an extremely important factor to determine the status of epithelial barrier. Higher acidity induces apoptosis and inhibits cell proliferation in colorectal carcinoma cell lines [[11](#_ENREF_12)]. The effects of feeding high concentrate diet for a long-term to lactating ruminants on the hindgut mucosa have never been explored. 2. For this purpose, we selected mid-lactating goat that is characterized by long lactating periods, stronger roughage resistance and stress resistance compared to other lactating ruminants. These animals recapitulate several key features of human hindgut epithelium, the most common type of monolayer structure. |
| Objectives | 4 | Therefore, the objective of this study was to evaluate the effects of feeding high concentrate diet for a long period on the histological structure and ultrastructure of the colonic mucosa, and the status of epithelial cells apoptosis in mid-lactating goats. |
| METHODS |  |  |
| Ethical statement | 5 | All animal experiments conformed to the “Guidelines on Ethical Treatment of Experimental Animals” (2006) No. 398 set by the Ministry of Science and Technology, China and “the Regulation regarding the Management and Treatment of Experimental Animals” (2008) No. 45 set by the Jiangsu Provincial People’s Government. |
| Study design | 6 | Two groups of 6 goats each were studied: LC. Low concentrate group fed a control diet comprising 65% forage and 35% mixed concentrate; HC. High concentrate group fed a high-grain diet containing 65% mixed concentrate and 35% forage.  In our experiment, twelve goats with similar body weight and similar milk yield were randomly allocated to LC and HC group.  In this study, n refers to number of animals (six animals each group).  Two weeks before the start of this experiment, goats were offered free access to a control diet comprising 65% forage and 35% mixed concentrate. After 2 weeks dietary adaptation, goats were randomly allocated to to LC and HC group. |
| Experimental  procedures | 7 | At the end of experiment, after an overnight fasting, all goats were euthanized with intravenous injection of Xylosol (0.5 mg/kg BW, xylazine; Ogris Pharme, Wels, Austria) and an intravenous injection of Release (50 mg/kg BW, pentobarbital; WDT, Garbsen, Germany) before slaughtered.  Immediately after slaughter at 8:00 AM, samples collecting including ruminal fluids, colonic digesta and mucosa tissues were finished within 30 min.  In the farm animal operation room, goat was euthanized and then put on an operating-table with 140 cm length and 65 cm width. After slaughter, samples were collected within 30 min.  Compared with other [routes of administration](http://en.wikipedia.org/wiki/Route_of_administration), the intravenous route (i.v.) is the fastest way to deliver fluids and medications throughout the body. |
| Experimental animals | 8 | Chinese dairy goats (49.7 ± 5.5 kg), mid-lactating stage, were included (n=6).  Twelve Chinese dairy-goat breeds (Guanzhong and Xinong Saanen) were obtained and acclimatized for at least 4 weeks. Health reports indicated that the goats were free of known viral, bacterial and parasitic pathogens. |
| Housing  and husbandry | 9 | Animals were housed in a temperature (22±2℃) and continuously illuminated stall.  All goats were allowed free access to water and housed in individual pens (1.0 m width and 1.5 m length). Both groups of goats were fed daily at 8:00 AM and 6:00 PM and were manually milked twice a day.  At the end of experiment, in order to reduce pain, all goats were euthanized with intravenous injection of Xylosol (0.5 mg/kg BW, xylazine; Ogris Pharme, Wels, Austria) and an intravenous injection of Release (50 mg/kg BW, pentobarbital; WDT, Garbsen, Germany) before slaughtered. |
| Sample size | 10 | Twelve mid-lactating goats were divided into two groups of six each. Animals of LC group received low concentrate group (65% forage and 35% mixed concentrate) and served as control, whereas HC group animals received high concentrate diet with 65% mixed concentrate and 35% forage.  Sample size calculations were performed in SPSS 11.0 for Windows (SPSS Inc., Chicago, IL, USA). Twelve goats with very close body weight and milk yield exhibited a baseline startle value deviating less than 2 SD from the whole group mean (a total of 30 animals were included.  The experiment was repeated, and data were calculated individually. |
| Allocating animals to experimental groups | 11 | For experiments using animals without surgery, animals with similar body weight and milk yield were allocated to two groups randomly. |
| Experimental outcomes | 12 | Three primary outcome measures were analyzed: morphology, ultrastructure and the apoptotic status of the colonic epithelium. In addition, four secondary outcome measures were evaluated: the level of volatile fatty acid (VFA) and LPS in ruminal fluid and colonic digesta, Caspase activities, gene and protein expression in the colonic mucosa. |
| Statistical methods | 13 | Data are presented as means ± SE. Statistical significance was assessed by the independent sample t-test using SPSS (SPSS version 11.0 for Windows; SPSS Inc., Chicago, IL, USA) software packages. Data was considered statistically significant when P < 0.05.  For each test, the experimental unit was an individual animal.    Test for normality was performed by the independent sample t-test. |
| RESULTS |  |  |
| Baseline data | 14 | The animals’ health status was monitored throughout the experiments by a health surveillance program according to Animal Ethics Committee at Nanjing Agricultural University guidelines. All goats were free of viral, bacterial, and parasitic pathogens. |
| Numbers analysed | 15 | Six goats in each group were included in each analysis. |
| Outcomes  and estimation | 16 | In accordance with the ARRIVE guidelines, we have reported measures of precision, confidence, and n to provide an indication of significance.  Twelve goats were randomly assigned to two groups (n = 6 per group).  Table 3: The effect of feeding LC or HC diet on rumen fermentation and colonic digesta parameters in goats at the time of slaughter. Data are presented as mean±SEM.   \| Item \| LC \| HC \| P-value \| \| --- \| --- \| --- \| --- \| \| Rumen fluid \|  \|  \|  \| \| Free LPS, EU/mL \| 25201±3398 \| 48395±4723 \| 0.004 \| \| Total VFA, mM \| 90.20±3.55 \| 116.37±8.14 \| 0.023 \| \| Colon digesta \|  \|  \|  \| \| Free LPS,EU/mL \| 22527±5325 \| 33613±5390 \| 0.182 \| \| Starch, mg/g mass \| 3.42±0.51 \| 4.56±0.93 \| 0.042 \| \| Total VFA, mM \| 44.68±3.35 \| 59.01±2.51 \| 0.007 \|   Fig.1 Caspase-3 and caspase-3/7 enzyme activity in the colonic mucosa. Data are presented as mean±SEM.  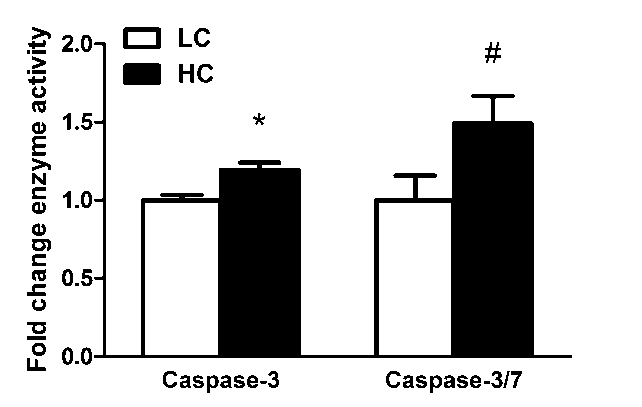  Fig. 2 Comparisons of morphological of the colonic mucosa between HC and LC goats. Colon section of LC group (A, scale bar = 100 μm); HC group (B, scale bar = 100 μm). Arrow indicates the damage of the colonic mucosa epithelium.  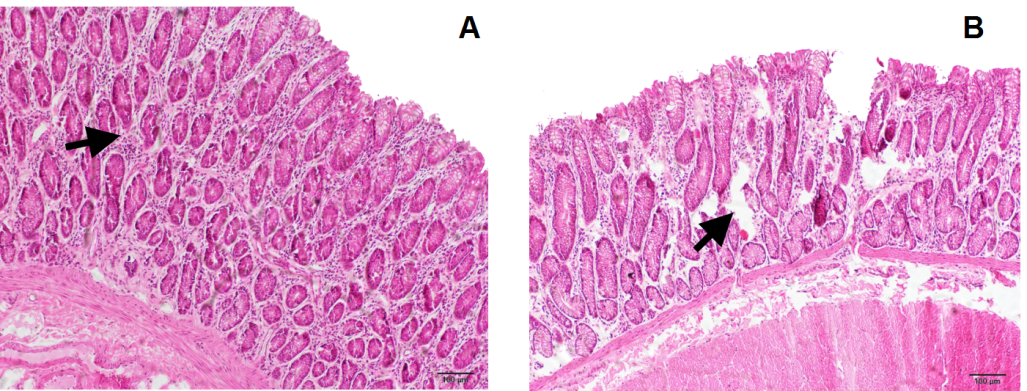  Fig. 3 Comparisons of ultrastructure of the colonic mucosa between HC and LC goats. Colon section of the (A) TJs of LC group; (B) nuclear of LC group; (C) mitochondria of LC group; (D) TJs of HC group; (E) nuclear of HC group; (F) mitochondria of HC group (transmission electron microscopy, × 10000). Arrow indicates the location of the TJs, nuclear or mitochondria (Scale bar=500 nm).  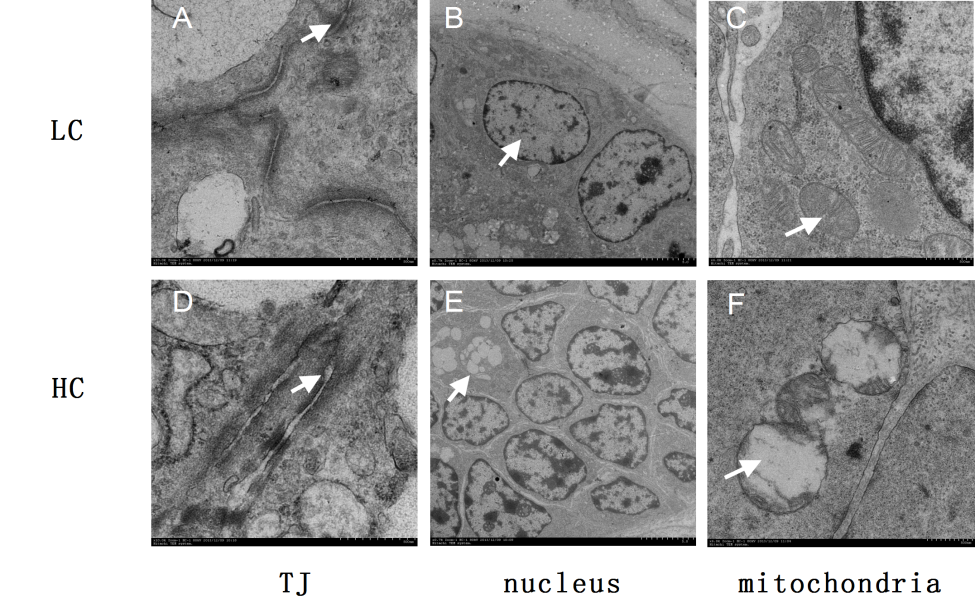  Fig. 4 Comparisons of TUNEL of the colonic mucosa between HC and LC goats. TUNEL-positive apoptotic cells were evaluated: colon section of the (A, scale bar = 20 μm) LC group; (B, scale bar = 20 μm) HC group. C: Analysis of the positive apoptotic cells. Data are presented as mean±SEM.  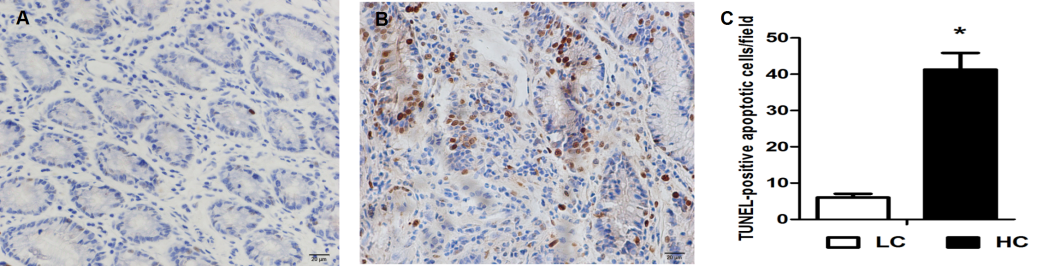  Fig. 5 Gene expression in the colonic mucosa. Data are presented as mean±SEM.  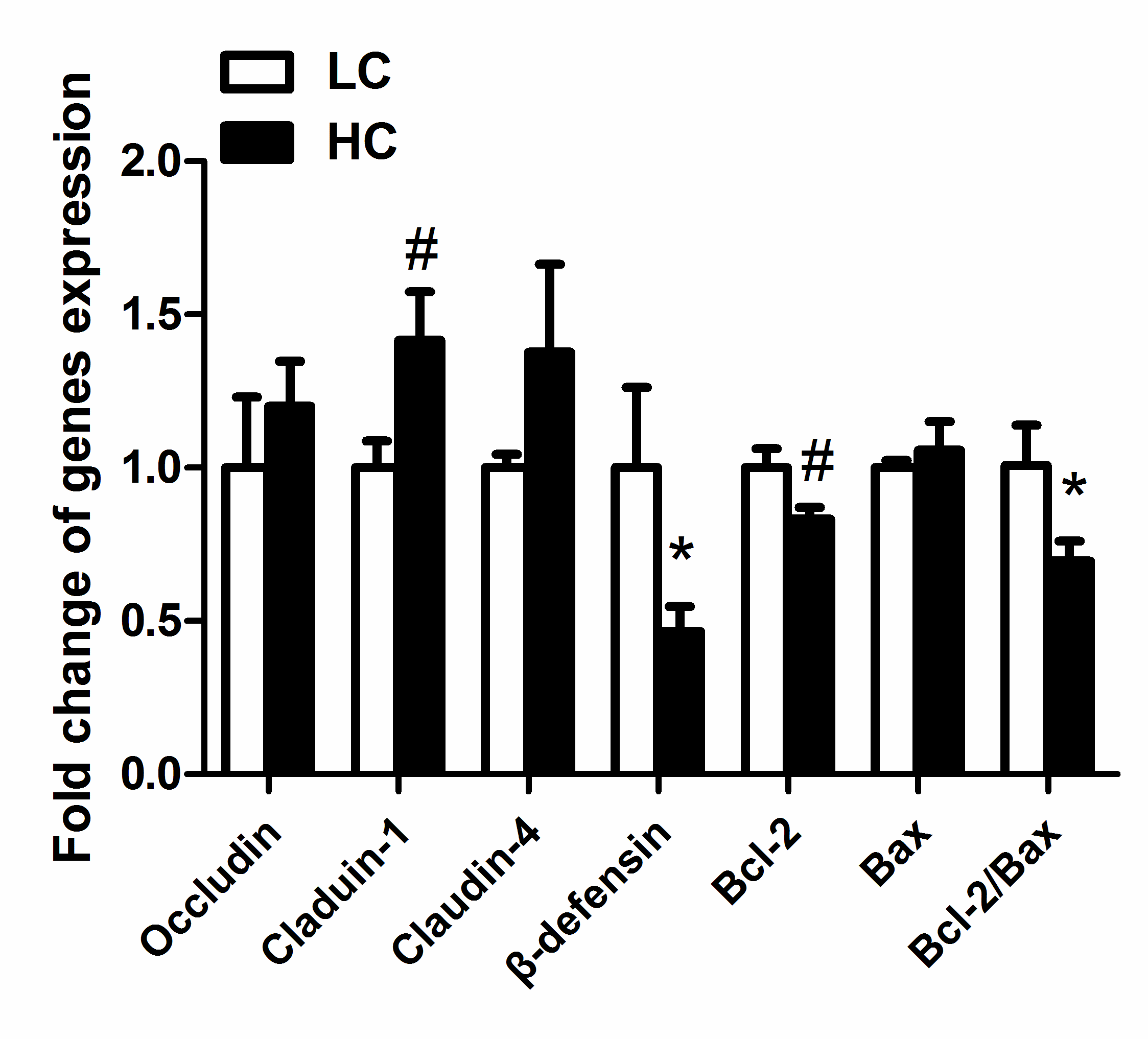  Fig. 6 Protein expression in the colonic mucosa. Data are presented as mean±SEM.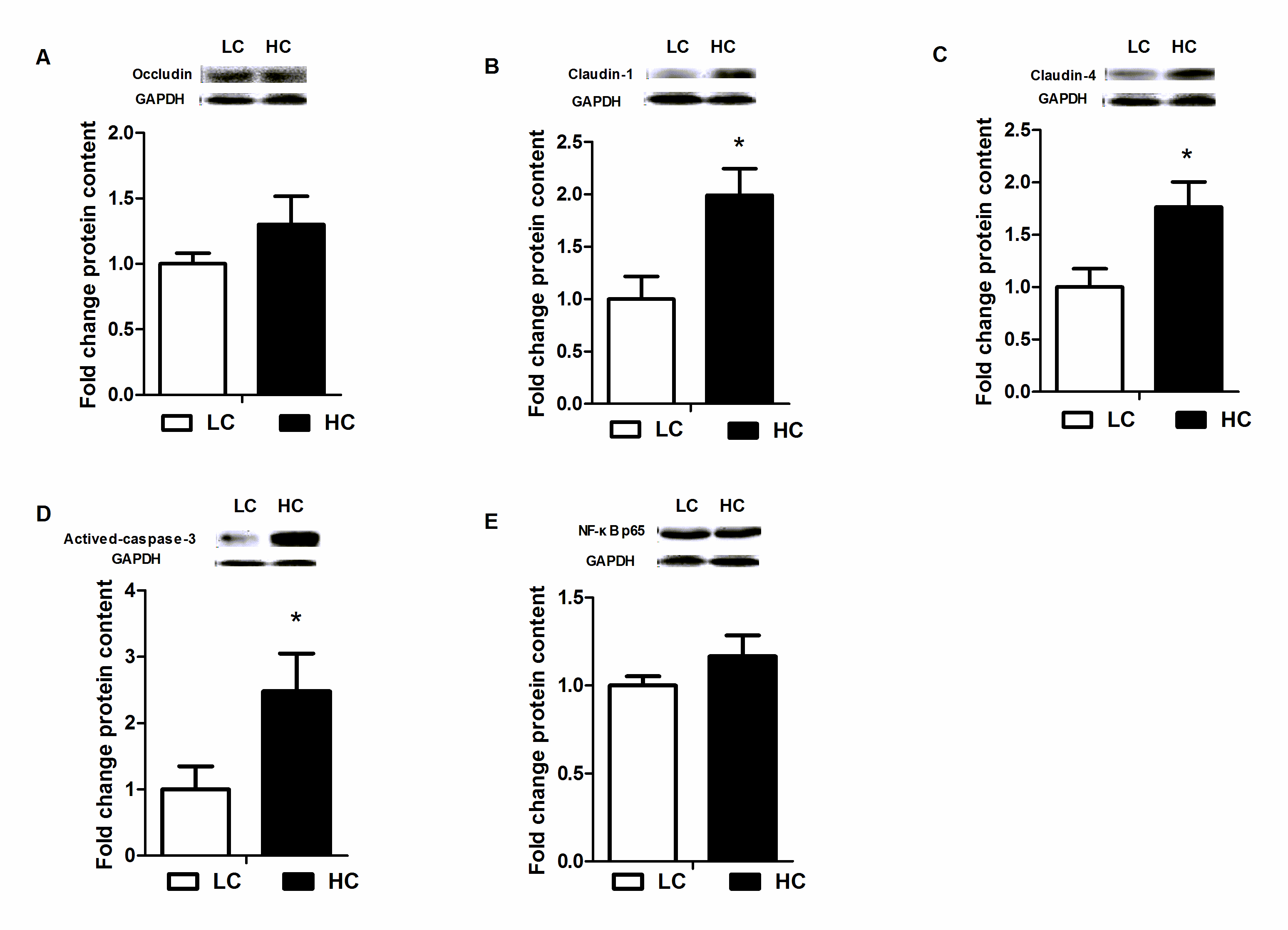 |
| Adverse events | 17 | There were no adverse events in all experiment animals. |
| DISCUSSION |  |  |
| Interpretation/ scientific implications | 18 | Feeding high-grain diets to lactating ruminants causes a high risk to damage the histological integrity and functions of ruminal epithelium in dairy cows [[3](#_ENREF_3), [4](#_ENREF_4)]. The aim of this study was to evaluate the effects of feeding high concentrate diet for a long period on the histological structure and ultrastructure of the colonic mucosa, and the status of epithelial cells apoptosis in mid-lactating goats. In our study, feeding HC diet to lactating goats for 10 wks resulted in severe damages to the colonic mucosa with widen TJs space and nuclear breakdown and mitochondrial swelling, which was associated with the activating of epithelial cells apoptosis.  The limitation of this study is the fact that the direct relationship between LPS and histological damages of colonic mucosa was not determined.  In our study, the small standard deviation (SD) indicates little individual deviation or a homogeneous group and shows potential for considerably reducing the number of animals used in tasks designed to detect the potential effects of high concentrate diet on the hindgut mucosa in ruminants. Based on previous report, we estimate that this could have been reduced to each group four animals [12]. |
| Generalisability/ translation | 19 | In our study, the results are important for facilitating the protection of chronic bowel disease in dairy ruminants especially in dairy cow. Given the similarity of the intestinal morphological structure especially the hindgut epithelium, knowledge of these targets is likely to help guiding the development and protection of inflammatory bowel disease (IBD) in human. |
| Funding | 20 | This work was supported by National Basic Research Program of China (Project No. 2011CB100802) and National Nature Science Foundation of China (Project No. 31272470) and A Project Funded by the Priority Academic Program Development of Jiangsu Higher Education Institutions (PAPD). |

**References**

1. Gaebel G, Bell M, Martens H: The effect of low mucosal pH on sodium and chloride movement across the isolated rumen mucosa of sheep. *Quarterly journal of experimental physiology* 1989, 74(1):35-44.

2. Plaizier JC, Krause DO, Gozho GN, McBride BW: Subacute ruminal acidosis in dairy cows: the physiological causes, incidence and consequences. *Veterinary journal* 2008, 176(1):21-31.

3. Emmanuel DG, Madsen KL, Churchill TA, Dunn SM, Ametaj BN: Acidosis and lipopolysaccharide from Escherichia coli B:055 cause hyperpermeability of rumen and colon tissues. *Journal of dairy science* 2007, 90(12):5552-5557.

4. Steele MA, Croom J, Kahler M, AlZahal O, Hook SE, Plaizier K, McBride BW: Bovine rumen epithelium undergoes rapid structural adaptations during grain-induced subacute ruminal acidosis. *American journal of physiology Regulatory, integrative and comparative physiology* 2011, 300(6):R1515-1523.

5. Graham C, Simmons NL: Functional organization of the bovine rumen epithelium. *American journal of physiology Regulatory, integrative and comparative physiology* 2005, 288(1):R173-181.

6. Penner GB, Steele MA, Aschenbach JR, McBride BW: Ruminant Nutrition Symposium: Molecular adaptation of ruminal epithelia to highly fermentable diets. *Journal of animal science* 2011, 89(4):1108-1119.

7. Stumpff F, Georgi MI, Mundhenk L, Rabbani I, Fromm M, Martens H, Gunzel D: Sheep rumen and omasum primary cultures and source epithelia: barrier function aligns with expression of tight junction proteins. *The Journal of experimental biology* 2011, 214(Pt 17):2871-2882.

8. Dong G, Liu S, Wu Y, Lei C, Zhou J, Zhang S: Diet-induced bacterial immunogens in the gastrointestinal tract of dairy cows: impacts on immunity and metabolism. *Acta veterinaria Scandinavica* 2011, 53:48.

9. Gressley TF, Hall MB, Armentano LE: Ruminant Nutrition Symposium: Productivity, digestion, and health responses to hindgut acidosis in ruminants. *Journal of animal science* 2011, 89(4):1120-1130.

10. Li S, Khafipour E, Krause DO, Kroeker A, Rodriguez-Lecompte JC, Gozho GN, Plaizier JC: Effects of subacute ruminal acidosis challenges on fermentation and endotoxins in the rumen and hindgut of dairy cows. *Journal of dairy science* 2012, 95(1):294-303.

11. Marques C, Oliveira CS, Alves S, Chaves SR, Coutinho OP, Corte-Real M, Preto A: Acetate-induced apoptosis in colorectal carcinoma cells involves lysosomal membrane permeabilization and cathepsin D release. *Cell death & disease* 2013, 4:e507.

12. [Giger-Reverdin S](http://www.ncbi.nlm.nih.gov/pubmed?term=Giger-Reverdin%20S%5BAuthor%5D&cauthor=true&cauthor_uid=24952476), [Rigalma K](http://www.ncbi.nlm.nih.gov/pubmed?term=Rigalma%20K%5BAuthor%5D&cauthor=true&cauthor_uid=24952476), [Desnoyers M](http://www.ncbi.nlm.nih.gov/pubmed?term=Desnoyers%20M%5BAuthor%5D&cauthor=true&cauthor_uid=24952476), [Sauvant D](http://www.ncbi.nlm.nih.gov/pubmed?term=Sauvant%20D%5BAuthor%5D&cauthor=true&cauthor_uid=24952476), [Duvaux-Ponter C](http://www.ncbi.nlm.nih.gov/pubmed?term=Duvaux-Ponter%20C%5BAuthor%5D&cauthor=true&cauthor_uid=24952476). Effect of concentrate level on feeding behavior and rumen and blood parameters in dairy goats: Relationships between behavioral and physiological parameters and effect of between-animal variability. [Journal of Dairy Science](http://www.ncbi.nlm.nih.gov/pubmed/24952476) 2014, 97(7):4367-4378.
